# An epidemiological study on anemia among institutionalized people with intellectual and/or motor disability with special reference to its frequency, severity and predictors

**DOI:** 10.1186/1471-2458-6-85

**Published:** 2006-04-03

**Authors:** Hiroko Ohwada, Takeo Nakayama, Nobuo Nara, Yuji Tomono, Keiko Yamanaka

**Affiliations:** 1 Department of Food Sciences, Ibaraki Christian University, 6-11-1 Ohmika Hitachi, Ibaraki, 319-1295, Japan; 2 Department of Health Informatics, Kyoto University School of Public Health, Yoshida-Konoe-cho, Sakyo-ku, Kyoto 606-8501, Japan; 3 Department of Laboratory Medicine, Tokyo Medical and Dental University, 1-5-45 Yushima, Bunkyo-ku, Tokyo 113-8519, Japan; 4 Ibaraki Prefectural Hospital of ASUNARO-NO-SATO, 1460 Sugizaki, Uchihara-machi, Higashiibaraki-gun, Ibaraki 319-0306, Japan

## Abstract

**Background:**

To examine the type, frequency, severity, and predictors of anemia and its relationship with co-morbid conditions among institutionalized people with intellectual and/or motor disability.

**Methods:**

We conducted a cross-sectional study at a public facility for people with intellectual and/or motor disability in Ibaraki prefecture, Japan. Health checkup data obtained in 2001 from 477 people with intellectual disability (male: 286, average age 40.6 ± 12.3; female: 191, average age 45.1 ± 11.6) were retrospectively reviewed.

**Results:**

The prevalence of anemia among male participants was higher than in female participants for each disability category (intellectual disability, 41.1%, 4.2%; cerebral palsy, 37.5%, 4.8%; Down's syndrome, 15.0%, 0%; severe motor and intellectual disabilities, 61.9%, 16.7%). Most participants with anemia (93.8 – 100%) showed a normocytic normochromic anemia pattern. Multivariate analysis revealed that factors related to an increase in frequency included sex (male), low body mass index (BMI), use of anticonvulsants or major tranquilizers, and a high zinc sulfate turbidity test (ZTT) value. No clinically diagnosed co-morbid condition was found to be related to the presence of anemia.

**Conclusion:**

A high frequency of mild normocytic normochromic anemia in institutionalized people with intellectual and/or motor disability was observed, particularly among males. Medications and chronic inflammation may increase the risk of anemia.

## Background

The 1992 American Association on Mental Retardation's (AAMR) definition and classification of mental retardation differs from the previous classification system in that: (a) a single diagnostic code of mental retardation is used if the individual meets the three criteria of age of onset (18 or under), significantly sub-average abilities in intellectual functioning, and related limitations in two or more adaptive skills areas; (b) the individual's strengths and weaknesses are described in reference to four dimensions: intellectual functioning and adaptive skills; psychological and emotional well-being; health, physical well-being, and etiology; and life activity environments; and (c) a profile of required support is developed across the four dimensions [1].

In recent years, interest in the quality of life of persons with intellectual disability has grown. However, epidemiological research on health and illness among people with intellectual disability has lagged when compared to similar research on healthy individuals.

Many persons with intellectual disability in Japan lead a collective lifestyle at a public facility as a community. These facilities provide an adequate diet, occupational rehabilitation and periodic medical checkups [2] and are thus fairly accommodable to conducting epidemiologic research on people with intellectual disability. In the past, nutritionists at these facilities have recognized a high frequency of anemia among institutionalized people with intellectual disability. Nevertheless, there continues to be a lack of epidemiological studies on the actual frequency, pattern, and predictors of anemia. The accumulation of relevant findings on this subject is strongly required. To this end, we conducted a cross-sectional study examining the type, frequency, severity, and predictors of anemia and its relationship with co-morbid conditions among institutionalized people with intellectual disability using blood profile tests conducted periodically at the facility.

## Methods

Participants in the present study consisted of 315 people with intellectual disability (male: 197, average age 40.3 ± 14.0; female: 118, average age 47.1 ± 11.4), 90 people with cerebral palsy (male: 48, average age 42.1 ± 10.7, female: 42, average age 43.0 ± 11.8), 33 people with Down syndrome (male: 20, average age 42.1 ± 9.5, female: 13, average age 47.5 ± 6.1), and 39 people with severe motor and intellectual disabilities (male: 21, average age 38.5 ± 10.6, female: 18, average age 35.2 ± 9.9) from a population of 477 persons with intellectual disability (male: 286, average age 40.0 ± 14.0; female: 191, average age 46.8 ± 11.6) institutionalized at a public facility in Ibaraki prefecture (109 km north of Tokyo, with a population of approximately 3 million), Japan. As of 2001, a total of 12,600 people in Ibaraki prefecture were recognized as intellectually disabled. This public facility houses more severely disabled persons. Over 95% of the residents were judged as having a severe level of intellectual disability. The subjects in the present examination were not diagnosed according to the AAMR's criteria; however, upon admission to the facility, subjects were diagnosed according to criteria similar to that of the AAMR, namely, the criteria of intelligence quotient (IQ), activities of daily life (ADL) and adaptive skills.

In Japan, periodical health checkups are provided for individuals in order to prevent lifestyle-related diseases including circulatory diseases. Recommended tests have been indicated for various health screenings. The present study retrospectively analyzed the existing health checkup data for each resident. The following information was collected at periodical health checkups administered in 2001: blood biochemical examinations, blood pressure, main disability, main diagnosis, IQs, and information regarding medications. The modified Binet Test for Japanese was used to calculate IQ at admission. According to the reference values of the institution, the definition of anemia was a hemoglobin (Hb) value ≤ 13.4 g/dl for males and ≤ 11.2 g/dl for females.

Patterns of anemia were classified with mean cellular volume (MCV) and mean corpuscular hemoglobin concentration (MCHC). Characteristics of anemic (normocytic normochromic anemia and anemia except normocytic normochromic anemia) and non-anemic individuals were analyzed separately. Multiple regression and logistic regression analyses using anemia as a dependent variable and c-reactive protein (CRP) and the zinc sulfate turbidity test (ZTT) as indices of inflammation estimated the following predictors of anemia: sex, age, body mass index (BMI), and the use of anticonvulsants and/or major tranquilizers, minor tranquilizers, iron replacement, or other medications (i.e. gastrointestinal drugs, drugs for a common cold, cardiovascular medication). ZTT is a turbidity test in which reagents are added to serum samples, and ZTT findings reflect the level of γ-globulins, in particular IgG.

Multivariate analysis was conducted using only three categories of people with intellectual disability. As the characteristics of people with severe motor and intellectual disability were very different from those of the other categories, the groups of patients with cerebral palsy and Down syndrome were combined. Assuming people with intellectual disability as a criterion, two dummy variables (G1 and G2) were used to express the influence of the groups of patients with cerebral palsy and Down syndrome on the presence of anemia (G1 and G2 = 0 for people with intellectual disability; G1 = 1, G2 = 0 for people with cerebral palsy; G1 = 0, G2 = 1 for people with Down syndrome). We reviewed the medical charts of each resident to gather information regarding physical conditions that can cause secondary anemia, such as thyroid dysfunction, hepatopathy, nephropathy, and gastrointestinal disease. Tests were not performed on all residents, but were performed on individuals for whom such tests were clinically required. The presence or absence of any of these diseases was taken into multivariate analysis in order to assess its relationship with anemia.

Furthermore, five men and five women were selected according to their Hb levels (the lowest) and were examined for their clinical characteristics. According to the reports from the registered dieticians at the institution, who determined the target food intake for each resident, all residents ate most of the foods that were prepared for them and primarily satisfied the target intake. Thus, the amount of food provided to each resident predominantly satisfies the Sixth Revised Japanese Nutritional Requirements.

All statistical analyses were performed using SPSS^® ^ver.12.0 statistical software (SPSS. Inc. Chicago, Illinois). Significance was established at the P = 0.05 level. The research protocol was approved by the Institutional Review Board at Miyagi Gakuin Women's University, which the first author (HO) was affiliated with at the time this study was conducted.

## Results

Severity of intellectual disability in mental retardation was defined as follows. Mild: IQ level 50–55 to approximately 70; moderate: IQ level 35–40 to approximately 50–55; severe: IQ level 20–25 to approximately 35–40; profound: IQ level below 20–25; and severity unspecified (when intellectual disability is strongly suggested but intelligence can not be tested by a standardized test) [3].

In Japan, public facilities house a larger ratio of individuals with severe intellectual disability than private facilities or individual residences. This was also true in our study. In most cases, subjects were institutionalized not on their will but rather as the result of an agreement between their guardian and the local government. The majority of participants obtained special education or did not receive any schooling due to their intellectual disability. Underlying diseases were unclear in the majority of cases. While diagnoses included cretinism, microencephaly, Sturge-Weber syndrome, fragile X syndrome, autism, tuberous sclerosis, Prader-Willi syndrome, and seizure, no trend towards a certain diagnosis was observed. Although a specific medical cause was not identified, in light of the dearth of epidemiological studies using large samples of people with intellectual disability institutionalized at the same facility, these findings provide valuable insight into the health of people with intellectual disability in Japan.

Tables 1, 2, 3, 4 show the characteristics of 315 institutionalized people with intellectual disability, 90 with cerebral palsy, 33 with Down syndrome, and 39 with severe motor and intellectual disabilities. Average BMI (kg/m2) was 21.5/22.0 (male/female) for people with intellectual disability; 20.7/21.6 for people with cerebral palsy; 22.2/24.0 for people with Down syndrome; and 14.3/15.1 for people with severe motor and intellectual disabilities. Concerning blood profiles, average red blood cell, white blood cell, Fe, total protein, albumin, total cholesterol, and high density lipoprotein cholesterol (HDLC) levels were all within the normal range for each category.

Average Hb level for people with severe motor and intellectual disabilities was 13.0 g/dl for males, which was lower than the normal range (13.5–17.6 g/dl). In contrast, average Hb levels for people in the other categories were all within normal ranges. The average level of ZTT for people with Down syndrome (male and female) was 15.5 units, which was slightly higher than the normal range (4–12 units). Average levels of CRP for people with intellectual disability (male), cerebral palsy (male and female), Down syndrome (male and female), and severe motor and intellectual disabilities (male and female) were higher than the normal range (0–0.30 units). Among people with intellectual disability, anemia was identified in 81 males (41.1%) and 5 females (4.2%). Among people with cerebral palsy, 18 males (37.5%) and 2 females (4.8%) were anemic. Among people with Down syndrome, 3 males (15.0%) and 0 females (0%) were anemic. Among people with severe motor and intellectual disabilities, anemia was identified in 13 males (61.9%) and 3 females (16.7%). The highest rate (143 people) of medication use was observed for males with intellectual disability. Nine (4.6%) males with intellectual disability took an iron replacement.

Tables 4, 5, 6, 7, 8 show the characteristics of anemic and non-anemic persons, including 314 institutionalized people with intellectual disability, 90 with cerebral palsy, 33 with Down syndrome, and 39 with severe motor and intellectual disabilities. Among anemic persons, 80 with intellectual disability (94.1%), 19 with cerebral palsy (100%), 3 with Down syndrome (100%), and 15 with severe motor and intellectual disabilities (93.8%) showed a normocytic normochromic anemia pattern. Average Fe levels of anemic people were lower than those of non-anemic people for all categories. Among people with intellectual disability, the level of Fe was within the normal range for 270 of the 314 people (86.0%) and was below the normal range for 44 of the 314 people (14.0%). Medication was more common in anemic people than in non-anemic people.

Estimates for predictors of anemia are shown in Table 9. Factors related to a decrease in frequency of anemia were a high BMI value and a diagnosis of Down syndrome, while factors related to an increase included sex (male), use of anticonvulsants or major tranquilizer, and a high ZTT value.

Table 10 shows conditions co-morbid with anemia and non-anemia cases among 477 institutionalized people with intellectual and/or motor disability. The most prevalent co-morbid conditions were gastro-intestinal disease (7.1%) followed by thyroid dysfunction (6.7%). When taking the presence of co-morbid conditions as explanatory variables in multiple regression or logistic regression analyses, no relationship with anemia was detected.

In case examinations of people whose Hb levels were the lowest among all subjects, the range of Hb was 9.4–10.7 g/dl for males and 9.8–10.6 g/dl for females. Routine health checkups did not identify any other unusual findings.

## Discussion

The findings of the present research indicated a high frequency of mild normocytic normochromic anemia among institutionalized people with intellectual and/or motor disability. Furthermore, the authors unexpectedly observed that anemia was much more prevalent in males than in females. This study also found that mild to moderate anemia occurred without a decrease in white blood cell count. In terms of subjective symptoms, it is difficult to determine the clinical importance of the anemia observed in the present study because the participants have intellectual and/or motor disability. However, Hb level has been widely accepted as an important index of nutritional status; therefore, it is of value to investigate the relationship between the anemia observed in the present research and the life prognosis of the participants.

To our knowledge, only a few case reports have discussed Down syndrome in investigations of anemia among people with intellectual disability [4,5]. As the literature on this epidemiological aspect is very limited, we expect that the present study will prompt similar studies in this field. We tested for the possibility of diet-induced anemia by observing the intake of periodic balanced meals [6]; however, this explanation did not appear to be plausible. In Japan, research on dietary assessment of people with intellectual and/or motor disability has just begun [2,6]; therefore, no reference data for the nutritional intake of people with intellectual and/or motor disability is available.

Among sample sizes large enough for multivariate analysis, results similar to those obtained in the analysis of the three combined categories (people with intellectual disability, cerebral palsy, and Down syndrome) were observed. For the other categories, the results obtained from multivariate analyses were not reliable due to small sample sizes.

The results of this cross-sectional study on predictors of anemia identified that a low BMI value, use of anticonvulsants and/or major tranquilizers, a high ZTT values and category of intellectual disability were predictors of an increased risk of anemia. However, the reason why anemia was more prevalent in males than in females remains unclear. A possible reason for this may be the reference values that were employed in the present study. The standard Hb value for anemia proposed by the WHO is <13.0 g/dl for males and <12.0 g/dl for females [7]. According to these standard values, the frequency of anemia was 26.2% for males and 19.5% for females with intellectual disability; 25.0% for males and 33.3% for females with cerebral palsy; 10.0% for males and 0% for females with Down syndrome; and 52.4% for males and 33.3% for females with severe motor and intellectual disabilities. Logistic regression analysis showed no correlation between sex and anemia, and the p value for ZTT became 0.11; however, no changes were observed for the other explanatory variables.

In a real clinical setting, the standard values proposed by the WHO are not the only available reference values. Individual facilities and standard textbooks provide a diverse range of reference values [8-10]. Therefore, in consideration of the unique characteristics of the participants in the present study, in order to determine the presence of anemia, we adopted the reference value of the facility where the study was conducted.

As BMI is an indicator of nutritional state, it has been found to be related with anemia [11]. In regards to medication, anemic subjects in the present study were likely to be taking carbamazepine (n = 25), valproic acid (n = 20), or levomepromazine (n = 14). Though previous studies have found that aplastic anemia is an adverse side effect of anticonvulsants (i.e. carbamazepine, Aethosuximide, felbamate, levetiracetam) and that thrombocytopenia is an adverse outcome of valproic acid, no studies have reported an association with anemia as observed in the present study [12,13].

However, one case report has described a possible relationship between hypoplastic anemia and chlorpromazine with a correlation to levomepromazine [14]. Sixty patients receiving long-term valproate (VPA) monotherapy were studied for hematologic side effects. Twenty developed at least one prominent hematologic abnormality. Thrombocytopenia and macrocytosis were the most common findings. Hematologic toxicity was never severe enough to discontinue therapy and always responded to small decrements in VPA therapy [15]. Our present study epidemiologically suggests that risk of anemia is related to medication. Nevertheless, given that data on medications were collected from medical records, this study is limited to an assessment of only currently prescribed medications. Accordingly, the duration of time medications were taken and whether subjects had to discontinue their medications for a period of time could not be considered.

Limitations in design also exist. As this study was cross-sectional, we can not deny the possibility that some individuals may stop their medication due to anemia. Judging from experience at this facility, however, medication regimen is not influenced by the onset of anemia. Accordingly, the present findings can not be used as an evaluation of risk. However, these findings did show a relation between the use of anticonvulsants and/or major tranquilizers and anemia with a limited possibility of severe anemia.

Given that a high ZTT value reflects IgG levels, the present findings suggest the possibility that chronic inflammation exists as an underlying factor. Chronic inflammation is well known to be a cause of normocytic normochromic anemia [16,17]. Given that CRP fluctuates even over short periods of time, inflammation observed with a high CRP value does not necessarily indicate that the inflammation is chronic. As the correlation between CRP and ZTT was weak (R = 0.12, P = 0.02), if chronic inflammation is indeed a cause of increased frequency of anemia among institutionalized persons, further studies assessing this possibility are required.

The examination of co-morbidity in the present study may indicate a relationship with chronic inflammation; however, no relationships were observed between co-morbid conditions and the presence of anemia. Another possible co-morbid condition could be the presence of periodontal disease and caries caused by poor oral hygiene; however, specific categories were not included in evaluations used at periodical health checkups. Therefore, we are currently analyzing data in reference to results obtained from dental health checkups.

Anemia may reflect many conditions such as gastrointestinal blood loss (these institutionalized residents would almost certainly be at high risk of Helicobacter pylori infection), gastroesophageal reflux (especially for those with severe disability), nutritional deficiencies, or chronic disease. However, data obtained from the medical records for the present analysis was limited to gastroesophageal reflux disease, gastro-intestinal disease, thyroid dysfunction, tonsillitis, liver disease, renal disease, parathyroid dysfunction, and sinusitis. Furthermore, diagnosis of these diseases was not standardized because of the nature of retrospective review. At least in the present study, as ascertained from medical charts, no clear correlation was observed between anemia and chronic diseases. The objective of the present study was to ascertain the frequency, severity, and predictors of anemia, not to clarify the cause of anemia; thus, further inquiry into the cause of anemia is beyond the scope of the present study.

When examining causes of anemia by MCV, low mean corpuscular volume implies the possibility of iron deficiency anemia, thalassemic disorders, anemia of chronic disease, sideroblastic anemia, copper deficiency, or zinc poisoning. Normal mean corpuscular volume implies the possibility of acute blood loss, iron deficiency anemia, anemia of chronic disease (eg, infection, inflammation, malignancy), bone marrow suppression, chronic renal insufficiency, or endocrine dysfunction (hypothyroidism, hypopituitarism). Increased mean corpuscular volume implies the possibility of valproic acid consumption, ethanol abuse, folic acid deficiency, myelodsplastic syndromes, acute myeloid leukemias, reticulocytosis, drug-induced anemia, or liver disease [15,18]. In the present study, 93.8–100% of anemia observed among people with intellectual and/or motor disability showed a normocytic normochromic anemia pattern. As multivariate analyses showed that high ZTT levels were associated with Hb levels or the presence of anemia, the anemia that was frequently observed in this study may be due to underlying chronic inflammation. Multivariate analyses were conducted after excluding people who were suspected of having iron deficiency anemia based on anemia patterns; however, the results were the same when these individuals were included. The results of multivariate analyses that did not include people who were suspected of having iron deficiency anemia also confirmed a correlation with ZTT.

This study highlights that medications and chronic inflammation should be considered in the evaluation of anemia among institutionalized people with intellectual and/or motor disability. The present study reviewed medical charts at one facility retrospectively; thus there are limitations in the generalization of the study results. Regarding the selection of the study population, random sampling of facilities across Japan is preferable. If national random sampling is not possible, it is necessary to accumulate data from other facilities or conduct multicenter studies. As epidemiological research on people with intellectual and/or motor disability is still in the developmental stage, further epidemiological research is needed on other health problems faced by people with intellectual and/or motor disability.

## Conclusion

A high frequency of mild normocytic normochromic anemia in institutionalized people with intellectual and/or motor disability was observed, particularly among males. Medications and chronic inflammation may increase the risk of anemia.

## Competing interests

We clarify that there is no conflict of interest regarding this manuscript.

## Authors' contributions

HO and TN conceived of the study, and participated in its design, conducted a survey, analyzed the data and wrote the manuscript. NN made some valuable suggestions on interpreting laboratory data and helped to draft the manuscript. YT and KY made some valuable suggestions in terms of clinicians who directly care for the participants of the present study and helped to draft the manuscript. All authors read and approved the final manuscript.

## Pre-publication history

The pre-publication history for this paper can be accessed here:



## Figures and Tables

**Table 1:** Characteristics in 315 institutionalized people with intellectual disability, Ibaraki prefecture, Japan.

	Male (n = 197)	Female (n = 118)
		
Age (years)	40.3 ± 14.0	47.1 ± 11.4
Intellectual quotients	29.0 ± 10.1 (n = 107)	28.8 ± 10.8 (n = 81)
Height (cm)	158.8 ± 9.6	146.4 ± 8.0
Weight (kg)	54.4 ± 11.5	47.1 ± 8.2
Body mass index (kg/m^2^)	21.5 ± 3.6	22.0 ± 3.8
Red blood cell (× 10^6^/μl)	4.5 ± 0.5	4.1 ± 0.3
White blood cell (× 10^3^/μl)	5.9 ± 1.8	5.6 ± 1.7
Hemoglobin (g/dl)	13.7 ± 1.2	12.6 ± 0.8
Anemic people (n)	81 (41.1%)	5 (4.2%)
Fe (μg/dl)	99.0 ± 39.6	103.8 ± 30.8
Total protein (g/dl)	7.2 ± 0.6	7.1 ± 0.7
Albumin (g/dl)	4.4 ± 0.4	4.3 ± 0.5
Total cholesterol (mg/dl)	164.7 ± 33.5	191.6 ± 39.4
HDL* (mg/dl)	54.3 ± 14.6	62.5 ± 16.2
ZTT* (unit)	9.6 ± 3.7	9.0 ± 3.5
CRP* (mg/dl)	0.6 ± 1.6	0.3 ± 1.1
Systolic blood pressure (mmHg)	118 ± 16 (n = 182)	114 ± 15
Diastolic blood pressure (mmHg)	75 ± 12 (n = 182)	72 ± 13
Numbers using certain medications (n)	143 (72.6%)	75 (63.6%)
Number on iron replacement (n)	9 (4.6%)	7 (5.9%)

*HDL: High density lipoprotein cholesterol, ZTT: Zinc sulfate turbidity test, C R P : C-reactive protein.

**Table 2:** Characteristics of 90 institutionalized people with cerebral palsy, Ibaraki Prefecture, Japan.

	Male (n = 48)	Female (n = 42)
		
Age (years)	42.1 ± 10.7	43.0 ± 11.8
Intelligence quotient	29.7 ± 11.7 (n = 24)	25.1 ± 16.8 (n = 22)
Height (cm)	154.4 ± 9.2	143.1 ± 7.5
Weight (kg)	49.6 ± 9.8	44.7 ± 10.0
Body mass index (kg/m^2^)	20.7 ± 3.0	21.6 ± 3.7
Red blood cells (× 10^6^/μl)	4.5 ± 0.5	4.1 ± 0.3
White blood cells (× 10^3^/μl)	5.7 ± 1.8	5.2 ± 1.4
Hemoglobin (g/dl)	13.7 ± 1.3	12.5 ± 0.8
Anemic cases (n)	18 (37.5%)	2 (4.8%)
Fe (μg/dl)	103.4 ± 44.6	99.1 ± 36.5
Total protein (g/dl)	7.3 ± 0.4	7.2 ± 0.5
Albumin (g/dl)	4.3 ± 0.3	4.4 ± 0.3
Total cholesterol (mg/dl)	168.8 ± 30.2	185.4 ± 36.0
HDL* (mg/dl)	53.7 ± 13.5	62.8 ± 16.2
ZTT* (unit)	10.6 ± 3.2	10.0 ± 4.0
CRP* (mg/dl)	0.8 ± 1.3	1.1 ± 0.2
Systolic blood pressure (mmHg)	117 ± 14 (n = 41)	114 ± 17
Diastolic blood pressure (mmHg)	77 ± 11 (n = 41)	75 ± 15
Cases using specific medications (n)	38 (79.2%)	30 (71.4%)
Cases on iron replacement (n)	2 (4.2%)	0 (0%)

*HDL: High density lipoprotein cholesterol, ZTT: Zinc sulfate turbidity test, CRP: C-reactive protein.

**Table 3:** Characteristics of 33 institutionalized people with Down's syndrome, Ibaraki Prefecture, Japan.

	Male (n = 20)	Female (n = 13)
		
Age (years)	42.1 ± 9.5	47.5 ± 6.1
Intelligence quotient	23.3 ± 6.3 (n = 13)	14.4 ± 12.8 (n = 11)
Height (cm)	146.3 ± 6.1	137.4 ± 5.5
Weight (kg)	47.7 ± 9.3	45.1 ± 7.5
Body mass index (kg/m^2^)	22.2 ± 3.5	24.0 ± 4.3
Red blood cells (× 10^6^/μl)	6.7 ± 10.2	4.2 ± 0.3
White blood cells (× 10^3^/μl)	6.2 ± 2.6	4.8 ± 1.3
Hemoglobin (g/dl)	14.3 ± 1.1	13.5 ± 0.7
Anemic cases (n)	3 (15.0%)	0 (0%)
Fe (μg/dl)	89.9 ± 25.9	101.0 ± 29.9
Total protein (g/dl)	7.4 ± 0.5	7.5 ± 0.3
Albumin (g/dl)	4.1 ± 0.3	4.2 ± 0.4
Total cholesterol (mg/dl)	176.2 ± 19.7	201.8 ± 34.8
HDL* (mg/dl)	49.7 ± 11.0	62.9 ± 14.5
ZTT* (unit)	15.5 ± 4.4	15.5 ± 5.0
CRP* (mg/dl)	0.6 ± 0.9	0.5 ± 1.0
Systolic blood pressure (mmHg)	110 ± 11 (n = 18)	101 ± 14
Diastolic blood pressure (mmHg)	73 ± 11 (n = 18)	65 ± 12
Cases using specific medications (n)	11 (55.0%)	5 (38.5%)
Cases on iron replacement (n)	0 (0%)	0 (0%)

*HDL: High density lipoprotein cholesterol, ZTT: Zinc sulfate turbidity test, CRP: C-reactive protein.

**Table 4:** Characteristics of 39 institutionalized people with severe motor and intellectual disabilities, Ibaraki Prefecture, Japan.

	Male (n = 21)	Female (n = 18)
		
Age (years)	38.5 ± 10.6	35.2 ± 9.9
Intelligence quotient	27.6 ± 14.6 (n = 11)	29.6 ± 11.3 (n = 5)
Height (cm)	151.4 ± 13.9	145.9 ± 8.4
Weight (kg)	33.2 ± 7.2	32.1 ± 7.5
Body mass index (kg/m^2^)	14.3 ± 2.4	15.1 ± 3.2
Red blood cells (× 10^6^/μl)	4.2 ± 0.4	4.1 ± 0.4
White blood cells (× 10^3^/μl)	5.4 ± 1.7	6.0 ± 2.4
Hemoglobin (g/dl)	13.0 ± 1.3	12.5 ± 1.4
Anemic cases (n)	13 (61.9%)	3 (16.7%)
Fe (μg/dl)	90.1 ± 29.2	105.1 ± 43.8
Total protein (g/dl)	7.2 ± 0.6	7.4 ± 0.5
Albumin (g/dl)	4.2 ± 0.4	4.1 ± 0.3
Total cholesterol (mg/dl)	181.3 ± 32.5	188.4 ± 35.0
HDL* (mg/dl)	55.4 ± 16.2	53.1 ± 13.0
ZTT* (unit)	9.8 ± 4.2	12.0 ± 5.2
CRP* (mg/dl)	0.6 ± 2.0	0.7 ± 2.0
Systolic blood pressure (mmHg)	106 ± 15	104 ± 25
Diastolic blood pressure (mmHg)	68 ± 12	62 ± 11
Cases using specific medications (n)	-	-
Cases on iron replacement (n)	-	-

*HDL: High density lipoprotein cholesterol, ZTT: Zinc sulfate turbidity test, CRP: C-reactive protein.

**Table 5:** Characteristics of anemic and non-anemic cases among 314a institutionalized people with intellectual disability, Ibaraki Prefecture, Japan.

	Anemic cases (n = 85)				Non-anemic cases (n = 229)	
			
	Normocytic normochromic anemia (n = 80)		Anemia except normocytic normochromic anemia (n = 5)			
				
	M (n = 75)	F (n = 5)	M (n = 5)	F (n = 0)	M (n = 116)	F (n = 113)
						
Age	37.8 ± 14.4	49.4 ± 16.7	30.4 ± 13.0	-	42.5 ± 13.4	47.0 ± 11.3
Body mass index (BMI)	20.5 ± 3.8	20.0 ± 2.9	22.2 ± 2.4	-	22.1 ± 3.4	22.1 ± 3.8
Fe (μg/dl)	90.4 ± 39.0	90.2 ± 22.9	84.2 ± 58.9	-	104.4 ± 37.8	104.4 ± 31.0
Medications (n)						
Anticonvulsants and/or major tranquilizers	58 (72.5%)		4 (80.0%)		92 (40.5%)	
Minor tranquilizers	18 (22.5%)		1 (20.0%)		31 (13.7%)	
Iron replacement	5 (6.3%)		0 (0%)		11 (4.8%)	
Other medications^b^	18 (22.5%)		2 (40.0%)		56 (24.7%)	
Users of any medication (n)	68 (85.0%)		5 (100%)		142 (62.6%)	
Non-medicated (n)	12 (15.0%)		0 (0%)		85 (37.4%)	

^a ^Missing data: one person.

^a^Medications except anticonvulsants and/or major tranquilizers, minor tranquilizers and iron replacement (i.e. gastrointestinal drug, cold drug, cardiovascular medication).

**Table 6:** Characteristics of anemic and non-anemic cases among 90 institutionalized people with cerebral palsy, Ibaraki Prefecture, Japan.

	Anemic cases (n = 19)				Non-anemic cases (n = 71)	
			
	Normocytic normochromic anemia (n = 19)		Anemia except normocytic normochromic anemia (n = 0)			
				
	M (n = 17)	F (n = 2)	M (n = 0)	F (n = 0)	M (n = 31)	F (n = 40)
						
Age	46.2 ± 10.7	41.0 ± 1.4	-	-	39.9 ± 10.2	43.1 ± 12.0
Body mass index (BMI)	19.8 ± 3.3	20.1 ± 2.1	-	-	21.2 ± 2.7	21.7 ± 3.7
Fe (μg/dl)	92.8 ± 36.5	84.5 ± 3.5	-	-	109.2 ± 48.0	99.9 ± 37.3
Medications (n)						
Anticonvulsants and/or major tranquilizers	12 (63.2%)		--		37 (52.1%)	
Minor tranquilizers	3 (15.8%)		--		13 (18.3%)	
Iron replacement	1 (5.3%)		--		1 (1.4%)	
Other medications^b^	6 (31.6%)		--		19 (26.8%)	
Users of any medication (n)	18 (94.7%)		--		50 (70.4%)	
Non-medicated (n)	1 (5.3%)		--		21 (29.6%)	

^a ^Medications except anticonvulsants and/or major tranquilizers, minor tranquilizers and iron replacement (i.e. gastrointestinal drugs, cold drugs, cardiovascular medication).

**Table 7:** Characteristics of anemic and non-anemic cases among 33 institutionalized people with Down's syndrome, Ibaraki Prefecture, Japan.

	Anemic cases (n = 3)				Non-anemic cases (n = 30)	
			
	Normocytic normochromic anemia (n = 3)		Anemia except normocytic normochromic anemia (n = 0)			
				
	M (n = 3)	F (n = 0)	M (n = 0)	F (n = 0)	M (n = 17)	F (n = 13)
						
Age	49.0 ± 5.2	--	--	--	40.9 ± 9.7	47.5 ± 6.1
Body mass index (BMI)	22.0 ± 3.4	--	--	--	22.2 ± 3.7	24.0 ± 4.3
Fe (μg/dl)	58.3 ± 11.9	--	--	--	95.4 ± 23.7	101.0 ± 30.0
Medications (n)						
Anticonvulsants and/or major tranquilizers	3 (100%)		--		3 (10.0%)	
Minor tranquilizers	3 (100%)		--		0 (0%)	
Iron replacement	3 (100%)		--		0 (0%)	
Other medications ^b^	2 (66.7%)		--		12 (40.0%)	
Users of any medication (n)	2 (66.7%)		--		14 (46.7%)	
Non-medicated (n)	1 (33.3%)		--		16 (53.3%)	

^a^Medications except anticonvulsants and/or major tranquilizers, minor tranquilizers and iron replacement (i.e. gastrointestinal drugs, cold drugs, cardiovascular medication).

**Table 8:** Characteristics of anemic and non-anemic cases among 39 institutionalized people with severe motor and intellectual disabilities, Ibaraki Prefecture, Japan.

	Anemic cases (n = 16)				Non-anemic cases (n = 23)	
			
	Normocytic normochromic anemia (n = 15)		Anemia except normocytic normochromic anemia (n = 1)			
				
	M (n = 12)	F (n = 3)	M (n = 1)	F (n = 0)	M (n = 8)	F (n = 15)
						
Age	36.3 ± 8.6	39.7 ± 2.9	62.0	--	38.8 ± 10.8	34.3 ± 10.6
Body mass index (BMI)	14.0 ± 2.8	12.5 ± 1.4	15.5	--	14.6 ± 1.8	15.6 ± 3.3
Fe (μg/dl)	76.0 ± 27.4	82.3 ± 15.6	88.0	--	111.5 ± 19.9	109.6 ± 46.5
Medications^a ^(n)						
Anticonvulsants and/or major tranquilizers	--		--		--	
Minor tranquilizers	--		--		--	
Iron replacement	--		--		--	
Other medications ^b^	--		--		--	
Users of any medication (n)	--		--		--	
Non-medicated (n)	--		--		--	

^a^Information on medication for people with severe motor and intellectual disabilities was not available.

^b ^Medications except anticonvulsants and/or major tranquilizers, minor tranquilizers and iron replacement (i.e. gastrointestinal drug, cold drug, cardiovascular medication).

**Table 9:** Predictors of anemia among 438a institutionalized people with intellectual and/or motor disability, Ibaraki Prefecture, Japan.

	Multiple regression		Logistic regression		
	Dependent variable:Hb		Dependent variable:anemia		
		
Predictor	B	*P*	OR	95%C.I.	*P*
		
Sex (Male = 1, Female = 0)	1.32	*<0.001*	13.77	6.02 – 31.50	*<0.001*
Age (46 ≦ = 1, 46 > = 0)	-0.15	*0.16*	1.42	0.81 – 2.49	*0.22*
Body Mass Index (21.3 ≦ = 1, 21.13 > = 0)	0.35	*0.001*	0.57	0.34 – 0.96	*0.03*
Medications					
					
Anticonvulsants and/or major tranquilizers	-0.44	*<0.001*	3.32	1.79 – 6.15	*<0.001*
					
Minor tranquilizers	0.05	*0.77*	0.77	0.38 – 1.59	*0.48*
Iron replacement	-0.13	*0.61*	1.88	0.54 – 6.54	*0.32*
Other medications ^b^	-0.08	*0.51*	1.59	0.85 – 2.95	*0.15*
CRP (0.31 ≦ = 1, 0.31 > = 0)	-0.22	*0.08*	1.42	0.82 – 2.45	*0.22*
ZTT (12.1 ≦ = 1, 12.1 > = 0)	-0.34	*0.01*	1.90	1.06 – 3.39	*0.03*
People with cerebral palsy ^c^	0.01	*0.94*	0.79	0.42 – 1.51	*0.48*
People with Down's syndrome ^c^	0.73	*0.001*	0.24	0.06 – 0.94	*0.04*

Adjusted R-square = 0.29

^a^Data set for analysis excluded 39 people with severe motor and intellectual disabilities from all 477 subjects.

^b^Medications except anticonvulsants and/or major tranquilizers, minor tranquilizers and iron replacement (i.e. gastrointestinal drugs, cold drugs, cardiovascular medication).

^c^Reference: people with intellectual disability

OR: Odds Ratio, C.I.: Confidence Interval,, C R P: C-reactive protein, ZTT: Zinc sulfate turbidity test.

**Table 10:** Conditions co-morbid with anemia among 477 institutionalized people with intellectual and/or motor disability, Ibaraki Prefecture, Japan.

Category	n	gastroesophageal reflux disease	gastro-intestinal disease	thyroid dysfunction	tonsillitis	liver disease	renal disease	parathyroid dysfunction	sinusitis
		
People with cerebral palsy	90	3	10	7	5	1	0	0	4
People with intellectual disability	315	5	20	15	9	12	4	2	10
People with Down's syndrome	33	1	2	0	0	0	0	0	2
People with severe motor and intellectual disabilities	39	1	2	10	1	2	0	0	0
		
Total	477	10	34	32	15	15	4	2	16

## References

[B1] Schalock RL, Stark JA, Snell ME, Coulter DL, Polloway EA, Luckasson R, Reiss S, Spitalnik DM (1994). The changing conception of mental retardation: implications for the field. Ment Retard.

[B2] Ohwada H, Nakayama T (2004). The utility of anthropometric assessment at institutions and schools for individuals with intellectual disabilities and/or motor disabilities: A nation-wide survey in Japan. J Nutr Sci Vitaminol.

[B3] American Psychiatric Association (2000). Diagnostic and Statistical Manual of Mental Disorders, (DSM-IV, text revision).

[B4] Pavithran K, Raji NL (2003). Aplastic anemia in Down's syndrome. Am J Hematol.

[B5] Hanukoglu A, Meytes D, Fried A, Rosen N, Shacked N (1987). Fatal aplastic anemia in a child with Down's syndrome. Acta Paediatr Scand.

[B6] Iwaoka H, Yokoyama T, Masayasu S, Fuchi T, Nakayama T, Tanaka H (1998). Characteristics of energy metabolism in males with mental retardation. J Nutr Sci Vitaminol.

[B7] http://www.who.int/nut/documents/ida_assessment_prevention_control.pdf.

[B8] Braunwald E, Fauci AS, Kasper DL, Hauser SL, Longo DL, Jameson JL (2001). Harrison's Principles of Internal Medicine.

[B9] Lee G, Richard (1993). Wintrobe's Clinical Hematolopgy.

[B10] Lawrence M, Tierney LM, McPhee SJ, Papadakis MA (2002). Current Medical Diagnosis & Treatment 2003: A Lange Medical Book.

[B11] Gray DS (1989). Diagnosis and prevalence of obesity. Med Clin North Am.

[B12] Lowenstein DH, Braunwald E, Fauci AS, Kasper DL, Hauser SL, Longo DL, Jameson JL (2001). Seizures and epilepsy. Harrison's Principles of Internal Medicine.

[B13] Brodie MJ, Dichter MA (1996). Antiepileptic drugs. N Engl J Med.

[B14] Kobashi H, Miyoshi I, Kimura I, Sakato J, Saino S, Suwaki H, Otsuki S (1979). A case of hypoplastic anemia presumably caused by chlorpromazine. Rinsho Ketsuek.

[B15] May RB, Sunder TR (1993). Hematologic manifestations of long-term valproate therapy. Epilepsia.

[B16] Jurado RL (1997). Iron, infections, and anemia of inflammation. Clin Infect Dis.

[B17] Bertero MT, Caligaris-Cappio F (1997). Anemia of chronic disorders in systemic autoimmune diseases. Haematologica.

[B18] http://www.utdol.com/application/image.asp?file=hema_pix/cause34.gif.

